# The Power of Equity: Addressing Disparities in Pain Management for Older Adults

**DOI:** 10.1093/geroni/igaf122.1527

**Published:** 2025-12-31

**Authors:** Tamara A Baker

**Affiliations:** The University of North Carolina at Chapel Hill, Chapel Hill, North Carolina, United States

## Abstract

Dr. Tamara A. Baker is a Professor in the Department of Psychiatry at the University of North Carolina at Chapel Hill. She is an appointed member of the US Department of Veterans Aﬀairs’ Geriatric and Gerontology Advisory Committee, a member of the National Ins tutes of Health’s Interagency Pain Coordina ng Commi ee, and a member of the Helping to End Addiction Long-Term (HEAL) Pain Strategic Plan’s Execu ve Commi ee. Dr. Baker is a member of Taylor & Francis’ editorial Advisory Board and Editor-in-Chief of Ethnicity & Health. She is formerly the Gerontological Society of America’s (GSA) Secretary and has served as Chair of the Commi ee on Minority Issues in Gerontology and Chair of the Behavioral and Social Sciences (BSS) section. She is a GSA Fellow, founder and co-convener of GSA’s Historically Black Colleges and Universi es (HBCU) Collaborative Interest Group, and appointed GSA Board of Governor’s Vice President (2025) and GSA President (2026). Her background in Gerontology, Psychology, and Biobehavioral Health has evolved into an active research agenda that focuses on understanding the behavioral and psychosocial predictors and outcomes of chronic pain and pain and symptom management among older adults from historically marginalized populations. She also examines health disparities and inequities in pain management, and access and availability to pain management resources among older adults. Broadly, her research includes health disparities and health equity, cultural diversity/sensitivity, and social determinants of health. Dr. Baker is recently funded in developing pipeline programs to address diversifying the aging research workforce.

